# Correlation of Chromosomal Instability, Telomere Length and Telomere Maintenance in Microsatellite Stable Rectal Cancer: A Molecular Subclass of Rectal Cancer

**DOI:** 10.1371/journal.pone.0080015

**Published:** 2013-11-21

**Authors:** Lisa A. Boardman, Ruth A. Johnson, Kimberly B. Viker, Kari A. Hafner, Robert B. Jenkins, Douglas L. Riegert-Johnson, Thomas C. Smyrk, Kristin Litzelman, Songwon Seo, Ronald E. Gangnon, Corinne D. Engelman, David N. Rider, Russell J. Vanderboom, Stephen N. Thibodeau, Gloria M. Petersen, Halcyon G. Skinner

**Affiliations:** 1 Division of Gastroenterology and Hepatology, Department of Internal Medicine, Mayo Clinic, Rochester, Minnesota, United States of America; 2 Department of Laboratory Medicine and Experimental Pathology, Mayo Clinic, Rochester, Minnesota, United States of America; 3 Department of Population Health Sciences, School of Medicine and Public Health, University of Wisconsin, Madison, Wisconsin, United States of America; 4 Department of Biostatistics and Medical Informatics, UW Carbone Cancer Center, University of Wisconsin, Madison, Wisconsin, United States of America; 5 Biostatistics and Informatics, Mayo Clinic, Rochester, Minnesota, United States of America; 6 Mayo Clinic Cancer Center, Mayo Clinic, Rochester, Minnesota, United States of America; 7 Health Sciences Research, Mayo Clinic, Rochester, Minnesota, United States of America; University of Nebraska Medical Center, United States of America

## Abstract

**Introduction:**

Colorectal cancer (CRC) tumor DNA is characterized by chromosomal damage termed chromosomal instability (CIN) and excessively shortened telomeres. Up to 80% of CRC is microsatellite stable (MSS) and is historically considered to be chromosomally unstable (CIN+). However, tumor phenotyping depicts some MSS CRC with little or no genetic changes, thus being chromosomally stable (CIN-). MSS CIN- tumors have not been assessed for telomere attrition.

**Experimental Design:**

MSS rectal cancers from patients ≤50 years old with Stage II (B2 or higher) or Stage III disease were assessed for CIN, telomere length and telomere maintenance mechanism (telomerase activation [TA]; alternative lengthening of telomeres [ALT]). Relative telomere length was measured by qPCR in somatic epithelial and cancer DNA. TA was measured with the TRAPeze assay, and tumors were evaluated for the presence of C-circles indicative of ALT. p53 mutation status was assessed in all available samples. DNA copy number changes were evaluated with Spectral Genomics aCGH.

**Results:**

Tumors were classified as chromosomally stable (CIN-) and chromosomally instable (CIN+) by degree of DNA copy number changes. CIN- tumors (35%; n=6) had fewer copy number changes (<17% of their clones with DNA copy number changes) than CIN+ tumors (65%; n=13) which had high levels of copy number changes in 20% to 49% of clones. Telomere lengths were longer in CIN- compared to CIN+ tumors (p=0.0066) and in those in which telomerase was not activated (p=0.004). Tumors exhibiting activation of telomerase had shorter tumor telomeres (p=0.0040); and tended to be CIN+ (p=0.0949).

**Conclusions:**

MSS rectal cancer appears to represent a heterogeneous group of tumors that may be categorized both on the basis of CIN status and telomere maintenance mechanism. MSS CIN- rectal cancers appear to have longer telomeres than those of MSS CIN+ rectal cancers and to utilize ALT rather than activation of telomerase.

## Introduction

Colorectal cancer (CRC) can be subdivided into tumors exhibiting chromosomal instability (CIN+) versus those with intact karyotype and chromosomal stability (CIN). Conventionally, only tumors with defective DNA mismatch repair (dMMR), otherwise known as microsatellite unstable tumors, (MSI(H)), were thought to be CIN-, and CIN+ tumors were thought to have intact dMMR and be microsatellite stable (MSS). However, several studies have demonstrated that up to 50% of MSS tumors are CIN-, and a significant, but smaller portion of MSI(H) colorectal tumors are CIN+ [1,2]. Although MSI(H) colorectal cancers are associated with a better prognosis than MSS tumors, recent studies have indicated that the level of chromosomal instability, rather than the microsatellite status, is the useful prognostic marker [3]. Specific phenotypes associated with the MSS CIN- subtype include poor tumor differentiation, mucinous histology and a lower rate of p53 mutations [1,4]. Moreover, CRC that arises at a younger age (<50 years old) is more likely to be MSS diploid, with diploidy being a surrogate measure of chromosomal stability. Chromosomally stable (CIN-) microsatellite stable (MSS) CRC is a relatively new classification of CRC that provides another framework of understanding the pathways underlying this cancer. 

Shortened telomeres have been linked to chromosomal instability (CIN) in the setting of age-related diseases associated with genetic disruption and carcinogenesis [5]. Telomeres are comprised of repeat TTAGGG sequences that protect the ends of linear chromosomes from being left vulnerable to damage, and dysfunctional shortening of telomeres has been implicated as the precursor to chromosomal instability. Critically short telomeres expose chromosomal ends, engage the DNA damage response, and precipitate end-to-end fusions and recombination events. Primary mammary epithelial cells with shorter telomeres are more frequently involved in mis-segregation events and exhibit not only chromosomal rearrangements but also numerical chromosomal changes [6], indicating that shorter telomere length may enable the development of CIN. Telomere length in DNA from MSS CIN- tumors has not been assessed. 

Telomeres may be lengthened by activation of the ribonucleoprotein reverse transcriptase named telomerase [7].. Telomerase activation is present in many cancers. Critically shortened telomeres normally initiate cell senescence and apoptosis and stop proliferation of cells containing significantly damaged DNA. Telomerase activation may effectively immortalize cancer cells by restoring enough telomere length to protect the numerically/ and structurally altered DNA from being neutralized by the DNA damage response and undergoing cell senescence. Another pathway of telomere maintenance through which telomeres may be rerouted from senescence is via alternative lengthening of telomeres (ALT), which is a homologous recombination based mechanism that uses a DNA template to preserve telomere length [8].

We sought to determine if distinct telomere maintenance pathway(s) would correlate with the presence or absence of CIN in MSS rectal cancer. To further clarify the phenomenon of MSS CIN- compared to CIN+ rectal cancer, we utilized array CGH (aCGH) to assess tumors for structural chromosomal and sub-chromosomal genetic alterations. 

As our previous study found that nearly 50% of young onset MSS CRC cases that arose in the proximal colon or in the rectum were diploid by flow cytometry [9], we studied only young age onset cases and focused on rectal cancers of similar stage. We measured telomere length in corresponding peripheral blood leukocyte, normal adjacent colon, and rectal cancer DNA and assessed the rectal cancer DNA for telomerase activation and ALT to determine if either of the two main mechanisms of telomere maintenance would correlate with the level of CIN in young onset MSS rectal cancer. 

## Methods

### Specimen selection and processing

This study was approved by the Mayo Clinic Institutional Review Board. We included rectal cancer patients from three sources: (1) Biobank for Gastrointestinal Health Research, a prospective repository comprised of annotated biospecimens from individuals with CRC who had evaluation at Mayo Clinic in Rochester, MN from April 2000 until August 2005 and who consented to be in the repository; (2) rectal cancer cases from a prospectively collected series of CRC patients who underwent surgical resection at the Mayo Clinic (Rochester, MN) from December 1995 until April 1997 [10] and (3) a prospective series of CRC patients who underwent surgery from 1987 through 1988 [11]. We selected 19 participants with MSS rectal cancers with onset at age ≤50 years old and which were either stage II (B2 or higher) or Stage III. Only participants with blood and/or normal colonic epithelium and tumor samples collected prior to any chemo or radiotherapy were selected for this study. DNA was extracted from peripheral blood leukocytes using the Gentra AutoPure salting out chemistry (Gentra, Minneapolis, MN) and quantified by UV absorbance. DNA quality was assessed by 260/280 optical density ratio. MSI status was measured using a combination of immunohistochemistry and PCR based analysis of MSI. Immunohistochemical staining for MLH1, MSH2, MSH6 and PMS2, and microsatellite instability testing were performed as previously described [12]. Microsatellite markers BAT26, D17S250; D5S346; ACTC, BAT40, BAT 25, BAT 34C4, D10S197, MYCL, and D18S55 were utilized to assess MSI status, and a tumor was called MSS if none of the markers showed MSI and all immunostains showed intact expression of MMR proteins [13].

### Tumor tissue processing and DNA extraction for array CGH

Frozen tumor specimens were used for the CGH array studies. Full thickness tumor and normal colonic epithelial tissues were excised from surgical specimens, snap frozen in liquid nitrogen and stored at -70°C. Tissue sectioning was performed in a cryostat at -20°C. Tumor tissue was macro-dissected to enrich for tumor density (>70% tumor nuclei). Briefly, a 6 micron thick frozen tumor section was stained with hematoxylin and eosin and marked as a guide slide by our pathologist (TCS) to identify regions containing ≥ 70% tumor nuclei. This slide was then used to identify the region of the corresponding frozen tumor block with only the area earmarked with 70% tumor density being sectioned to be used as the tumor tissue source for DNA. Accompanying normal colonic mucosa, a minimum of 8 cm from the tumor margin, was microdissected for epithelial tissue. Sections were placed in extraction buffer and genomic DNA was extracted from tumor or normal colon epithelium DNA by Phenol/ Chloroform extraction and quantified by UV absorbance, and DNA quality was assessed by 260/280 optical density ratio. In all cases, DNA was extracted from chemoradiotherapy naïve rectal cancer.

### Array CGH

aCGH was performed using the Spectral Chip™ 2600 (Spectral Genomics, Houston, TX) which contains 2600 BAC clones spotted in duplicate. Both forward and reverse labeling experiments were done for each tumor samples. Labeling and hybridization were performed according to the manufacturer's protocol for the Spectral Chip™ 2600. Ultra-pure deionized H_2_O was used for the preparation of all reagents; Promega Male Genomic DNA (Madison, WI) was used as reference DNA; dye-reversal experiments in which 2 microarrays were performed for each specimen with reciprocal labeling of the test and reference DNA. The test and reference DNA were random primed labeled by combining 2 μg genomic DNA and ddH_2_O to a total volume of 50 μL and sonicating in an inverted cup horn sonicator to obtain fragments 600 bp to 10 kb in size. DNA cleanup was performed with Zymo's Clean-up Kit (Orange, CA) according to protocol. The elutant was split equally between 2 tubes and, to each, 20 μL 2.5× random primers from Invitrogen's (Carlsbad, CA) BioPrime DNA Labeling Kit was added, mixed well, boiled 5 minutes, and immediately placed on ice for 5 minutes. To each was added 0.5 μL Spectral Labeling Buffer (Spectral Genomics), 1.5 μL Cy3-dCTP or 1.5 μL Cy5-dCTP respective to each dye-reversal experiment (PA53021, PA55021; Amersham Pharmacia Biotech, Piscataway, NJ), and 1 μL Klenow fragment (BioPrime DNA Labeling Kit; Invitrogen). The contents were incubated for 1 hour at 37°C. The tubes were then reheated to boiling for 5 minutes and returned to the ice for 5 minutes. Another 5 ul aliquot of the labeling buffer was added to the mixture and the tubes were incubated at 37°C for another hour. At the end of the second hour, adding 5 μL 0.5 M EDTA pH 8.0 and incubating the tubes in a dry bath for 10 minutes at 72°C stopped the labeling reaction. Sample and control DNA mixtures were then combined into the sample tube, 45 ul of Hyb I (blocking DNA and salmon sperm carrier DNA) were added to each tube and 5 M NaCl and 100% isopropanol were added to the tubes to precipitate the DNA. DNA pellets were washed with 70% ethanol X1 and then dried in the dark. After re-hydrating the pellets with water, HYBII (hybrisol) was added to the mixture and the DNA was denatured for ten minutes at 72°C then pre-hybridized for 30 minutes before being placed on the array and covered with a 24X60mm cover slip. Slides were incubated overnight in a 37°C oven.

Post-hybridization washes were performed with each slide in individual deep Petri dishes in a rocking incubator. After removing the coverslip, the slides were briefly soaked in 0.5% SDS at room temperature. Each slide was then transferred to 2XSSC, 50% de-ionized formamide pH 7.5 for 20 minutes; then 2XSSC, 0.1% IGEPAL CA-630 pH 7.5 for 20 minutes followed by 0.2XSSC pH 7.5 for 10 minutes, each pre-warmed to 50°C and agitated in an incubator at 50°C. Finally, each slide was briefly rinsed in two baths of room temperature ddH_2_O and immediately blown dry with compressed N_2_ and scanned. 

### Array data analysis

Scanning was performed with Axon's GenePix 4000B microarray scanner (Molecular Devices, Sunnyvale, CA) and the images were analyzed with Genepix Pro 3.0 to prepare data files for plot analysis with Spectralware software V2.2. (Spectral Genomics, Houston, TX). Spots were defined by the automatic grid feature of the software and manually adjusted when necessary. Spots showing no signal or obvious defects were excluded from the data analysis, local background was subtracted, and total intensities, as well as the fluorescence intensity ratios of the two dyes, were calculated for each spot. Plot analysis was performed using the online accessible version of SpectralWare software (Spectral Genomics) using global mean normalization of the data. In addition, the datasets were analyzed using Microsoft Excel. After performing global mean and global median normalizations, the mean ratios of four fluorescent signals (two signals from the duplicated clone on the array and two signals from the color reverse experiment) for each clone were calculated. All analysis was done on log_2_ ratios. To reduce false positive results, clones showing test/reference ratio value higher than 1.2 were considered gained and clones showing test/reference ratio value lower than 0.8 were considered lost, but only if the results of all four fluorescent signals were consistent. Clones were excluded from analysis if the ratio values of the four hybridized spots of each clone exceeded the threshold values (0.8–1.2) in a non-concordant matter. 

### Quantitative PCR assessment of telomere length

Telomere length was assessed in tumor DNA from all rectal cancer cases and from those with adequate normal colonic epithelium DNA using DNA as prepared for the array CGH assay [14]. In a portion of cases, PBL DNA was available and extracted using the Gentra AutoPure chemistries as described above. 

Two master mixes of PCR reagents were prepared, one with the T primer pair, the other with the S primer pair. Fifteen microliters of the T master mix were added to each sample well, control well and standard curve well of the first plate and 15 µl of the S master mix were added to each sample well, control well and standard curve well of the second plate. 

For each sample assayed, three identical 5 µl aliquots of the DNA sample (15 ng/aliquot) were added to plate 1 and another 3 aliquots were added to the same well position in plate 2. For each standard curve, one reference DNA sample was serially diluted in TE by 1:2 fold per dilution to produce six concentrations of DNA ranging from 0.78 to 25ng/µl. 

Five microliters of each concentration was distributed to the standard curve wells on each plate. The plates were then sealed with a transparent adhesive cover, centrifuged briefly at 800 g and transported on ice to the ABI 7900HT instrument for analysis. 

The T and S PCRs were prepared identically with the exception of the oligonucleotide primers. The final concentrations of the reagents in the PCR were 20 mM Tris-HCl, 0.2 mM each dNTPs, 2.0 mM MgCl², 1%DMSO, 150 nM ROX dye, 0.2X Sybr Green I (Molecular Probes), 5 mM DTT, 1.25 U AmpliTaq Gold DNA polymerase (Applied Biosystems). The final telomere primer concentrations was tel 1b, 600 nM; tel 2b 900 nm. The control gene (B2-globin on chromosome 11) concentrations was HBB1 300 nm; HBB2 700 nm. The primer sequences (written 5´ - 3´) was tel 1b: CGGTTTGTTTGGGTTTGGGTTTGGGTTTGGGTTTGGGTT; 

tel 2b: GGCTTGCCTTACCCTTACCCTTACCCTTACCCTTACCCT; 

HBB1: GCTTCTGACACAACTGTGTTCACTAGC; 

HBB2: CACCAACTTCATCCACGTTCACC. 

All PCRs were performed on the ABI Fast Real-Time 7900HT (Applied Biosystems, Foster City, CA). The thermal cycle conditions for both primers pairs began with a 95°C incubation for 10 minutes to activate the AmpliTaq Gold DNA polymerase. For telomere PCR, this was followed by 40 cycles of 95°C for 15 seconds, 54°C for 2 minutes. For the HBB PCR, this was followed by 40 cycles of 95°C for 15 seconds, 58°C for 60 seconds, and 72°C for 30 seconds. The data was then analyzed with the ABI SDS software to generate the standard curve for each plate. 

### Methodology for ABI 7900HT using SYBR Green Dye 1

The SYBR Green 1 Double-Stranded Binding Dye binds non-specifically to dsDNA and generates an excitation emission profile. For quantitative PCR, SYBR Green 1 was used with a passive reference dye (ROX). The 7900HT instrument analyzed the cycle to cycle change in fluorescence signal as a result of amplification during a PCR. During normal amplification of the PCR product three regions characterize the progression of the PCR. 

### Telomere length analysis

Telomere lengths measured by PCR are often reported as a ratio of the telomere (T) and standard gene (S) measurements (T/S). As base pair length is intuitively easier to understand, the ratios were transformed into base pair length using a previously validated formula reported with the technique by Cawthon described above [14]. The results of statistical tests comparing groups are the same whether the T/S ratios or telomere length are used. As base-pair length is intuitively easier to understand, Southern blots were performed to determine telomere restriction fragment (TRF) length on a subset of participants (n=16) and a linear regression was used to compare TRF length to the T/S ratio, resulting in the following equation: bp = [T/S]*1470.8+7674.5 where 1470.8 represents the slope of the line comparing the relative T/S ratio to the measurement of telomere length in base pairs by the mean TRF and 7674.5 is the y intercept. 

### p53 mutation analysis

Adequate quantities of tumor DNA were available from 17 of the 19 tumors to test for somatic p53 mutations. 

PCR amplification was performed in a total volume of 12.5 μl containing 5 ng of genomic DNA, each primer at 0.2 mM, dNTP at 0.2 mM, 2.0 mM MgCl_2_, 0.5 U of Taq polymerase (AmpliTaq Gold, Applied Biosystems, CA) with PCR reaction buffer provided by the manufacturer. Denaturing high-performance liquid chromatography (DHPLC) analyses followed by direct sequencing of the PCR products were performed as described previously (14).

The coding sequence and the splice junction sites of *TP53* exons 5-8 were PCR amplified using 4 pairs of intronic primers as follows:

Exon5F 5' GCC GTC TTC CAG TTG CTT 3'
Exon5R 5' CAA CCA GCC CTG TCG TCT CT 3'
Exon6F 5' GGG GCT GGA GAG ACG ACA 3'
Exon6R 5' TCC TCC CAG AGA CCC CAG TT 3'
Exon7F 5' CTT GCC ACA GGT CTC CCC AA 3'
Exon7R 5' AGG GGT CAG CGG CAA GCA GA 3'
Exon8F 5' AAA TGG GAC AGG TAG GAC 3'
Exon8R 5' AAG TGA ATC TGA GGC ATA AC 3'

All PCR reactions were carried out in a 25 µl reaction volume consisting of 10X PCR buffer II (Applied Biosystems, Foster City, CA), 2 mM MgCl2, 2.5 mM of each dNTP, 10 µM of each primer, 0.75 units of Taq AmpliGold DNA polymerase (Applied Biosystems, Foster City, CA), and 10 ng of template DNA. PCR was performed using a Tetrad thermal cycler (MJ Research, Waltham, MA) with the following conditions: initial denaturation at 94°C for 9 minutes, followed by 35 cycles at 94°C for 30 seconds, (58°C for 30 seconds exons 5,6,7) and (55°C for exon 8), 72°C for 45 seconds, and final extension at 72°C for 10 minutes. The PCR products were run on a 2% agarose check gel. PCR products were prepared for DNA sequencing as followed: 5 µl of PCR product with 1 µl each exonuclease and 10X shrimp alkaline phosphate TUF buffer then ran on a Tetrad thermal cycler with the following conditions: 37°C for 15 minutes, 80°C for 15 minutes. 1.6pmol of primer along with the template were sequenced on the ABI PRISM™ 3700 DNA Analyzer (Perkin Elmer Applied Biosystems, Foster City, CA 94404) in the Core Facility. Mutation Surveyor Software (SoftGenetics LLC, State College, PA) was used to analyze the results.

### Assessment of telomerase activation in CRC

Telomerase activity was measured using the TRAPeze^TM^ telomerase detection kit. (Chemicon International). Macrodissected tumor tissue was homogenized in 200 μl of 1x CHAPS lysis buffer, then incubated for 30 min on ice and centrifuged at 4°C and 12000 g for 20 minutes. To 2.0 μl of this supernatant, 5.0 μl of 10X TRAP buffer, 1.0 μl of a mixture of 10x d-NTPs, 1.0 μl of TS primer mix and 0.4 μl of TaqDNA polymerase was added to a total volume of 50 μl. Telomere extension at 30°C for 10 minutes was followed by 30 PCR cycles at 94°C for 30 seconds and 60°C for 30 seconds. 10 μl of the PCR product was run on a 12% polyacrylamide gel and stained with SYBR^TM^ gold (Molecular Probes, Eugene, OR). The gel was photographed with a UV transilluminator and ladder. Telomerase activity was expressed as the band ratio between the ladder of the PCR product and internal control.

### Assessment of ALT

Telomeric C-Circle DNA are partially single-stranded telomere DNA circles specific to cells exhibiting ALT [15]. C-circles were assessed in tumor DNA using isothermic amplification of C-circle complementary strand and hybridization with ^32^P-(CCCTAA)_3_ probe by Capital Biosciences (Capital Biosciences, Maryland, U.S.A.). A DNA sample was called ALT+ if C-circles were detected. C-circles are extrachromosomal telomeric DNA comprised of partially double stranded telomeric circles with a partially double stranded telomeric DNA with a continuous C rich strand and incomplete G rich strand. C-circles are strongly associated with the presence of ALT [15].

### Analytic approach

To establish CIN status based on chromosomal gains or losses, data were analyzed in R using the aCGH package from Bioconductor [16]. 

Filtering was conducted to remove unmapped clones, clones mapping to the Y chromosome, and clones missing in more than 25% of subjects. We then imputed missing observations using a LOWESS approach. The fraction of participants with gains or losses of each clone was plotted by chromosome number and location, by CIN status (Figure S1).

In addition, the Wilcoxon test was used to evaluate the difference in CRC tumor telomere length between: 1) CIN+ and CIN- rectal cancer , 2) ALT+ and ALT- samples, and 3) telomerase+ and telomerase- samples [17]. Chi-squared tests or Fisher’s exact test were used to evaluate the association between ALT status and CIN status, and between telomerase activation status and CIN status. The spearman correlation between rectal cancer tumor telomere length and the percentage of clone with gains or losses was calculated.

## Results

### Array CGH evinced extensive variation in DNA copy number changes

We studied 19 young-onset MSS rectal cancers for evidence of CIN using aCGH comprised of roughly 3000 BACs. We demonstrated that there are MSS rectal tumors that have very low levels of DNA copy number changes. Six tumors (32%) had low levels of chromosomal disruption with <1% to 17% of the clones having DNA copy number changes and were classified as CIN- by aCGH. Thirteen tumors (68%) had >20% of clones showing DNA copy number changes and were classified as the CIN+ group. MSS CIN- tumors had 0 to fewer than 5 chromosomal arms that were completely gained or lost, compared to the CIN+ group which had 8 to 18 chromosomal arms showing complete gain or loss (Figure S2).

### Fraction of gains or losses in CIN+ and CIN- rectal cancer

 The fraction of participants with gains and losses per chromosomal region for subjects with CIN+ rectal cancer compared to those with CIN- rectal cancer are displayed in Figure S1. Differences in gains or losses were observed throughout the genome, with the most pronounced differences in chromosomes 13, 17 and 18. CIN+ rectal cancer had more gains/losses than CIN- rectal cancer (mean: 33.1 vs 9.7, p=0.0007, Table 1). 

**Table 1:** Characteristics of CIN+ and CIN- rectal cancer.

	**CIN+**	**CIN-**	
	**Mean (SD) or %**	**Mean (SD) or %**	**p-value**
Fraction gains/losses, mean (SD)	33.08 (8.76)	9.66 (7.92)	0.0007
Telomere length, mean (SD)	8211 (308)	9178 (1396)	0.0066
Telomerase			0.0949
Yes	80.0%	25.0%	
No	20.0%	75.0%	
ALT			0.2335
Yes	50.0%	100.0%	
No	50.0%	0.0%	
p53			0.3348
Yes	36.4%	66.7%	
No	63.6%	33.3%	

Colorectal cancer tumors evaluated with array CGH were classified as 1) CIN- if <20% of clones showed DNA copy number changes or as 2) CIN+ if ≥ 20% of clones showed DNA copy number changes. CIN+ CRC had a higher number of cases that exhibited gains and losses per chromosomal region compared to those of CIN- CRC. CIN+ CRC has significantly shorter tumor telomeres (8211 bp) than CIN- CRC (9178 bp; p=0.0066). CIN+ tumors were more likely to show activation of telomerase than were CIN- CRC, though there was no correlation with the CIN status of a tumor and how often a tumor used alternative lengthening of telomeres as a telomere maintenance mechanism (p=0.2335). The presence of p53 mutations did not correlate with the CIN status of the tumor (p=0.3348).

### Telomere length in CIN+ and CIN- rectal cancer

**Figure 1:**
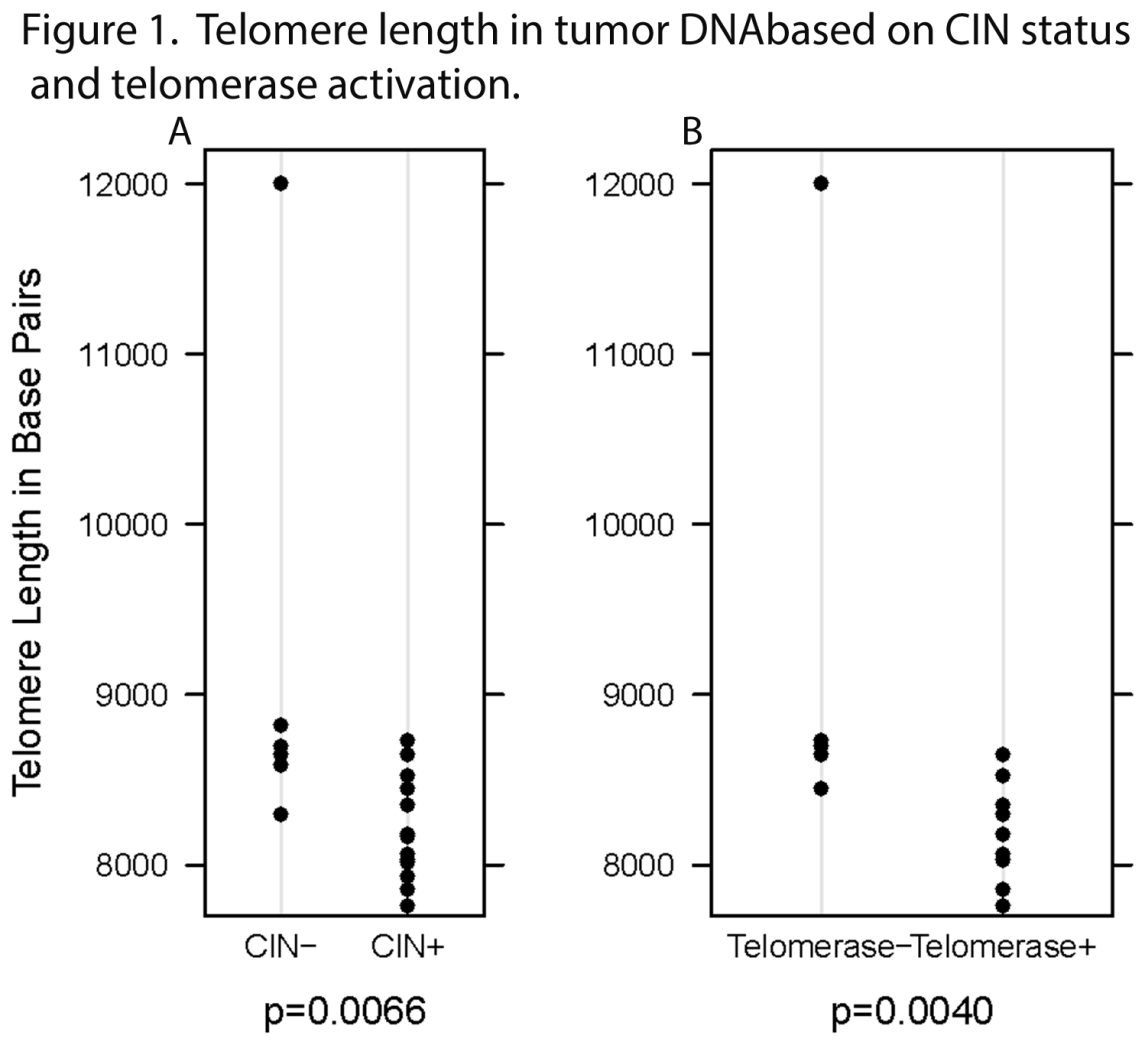
Telomere length in tumor DNA based on CIN status and telomerase activation. Panel A: The telomere length of tumor DNA in chromosomally stable (CIN-) rectal cancer is significantly longer than that of chromosomally unstable (CIN+) rectal cancer (p=0.0066). CIN status is determined as CIN- if from <20% of clones show DNA copy number gains or losses. Rectal cancer is CIN+ if more than 20% of clones have gains or losses. Panel B: Activation of telomerase (Telomerase +) in rectal cancer correlates with shorter tumor telomere length than in tumors that do not utilize telomerase (Telomerase -) as a telomere maintenance mechanism (p=0.0040).

Rectal cancer telomere length was significantly associated with CIN status, such that CIN- rectal cancer had longer telomeres than CIN+ rectal cancer (p=0.0066, Figure 1). Telomere length was also significantly correlated with the percentage of clones with gains or losses (r=-0.50, p=0.0310). 

Among the CIN- tumors, telomeres were longer in the tumor DNA compared to their corresponding normal epithelial cell DNA. In contrast, telomeres in CIN+ tumor DNA were shorter than those in corresponding normal epithelium DNA. (p=0.0039). 

**Figure 2:**
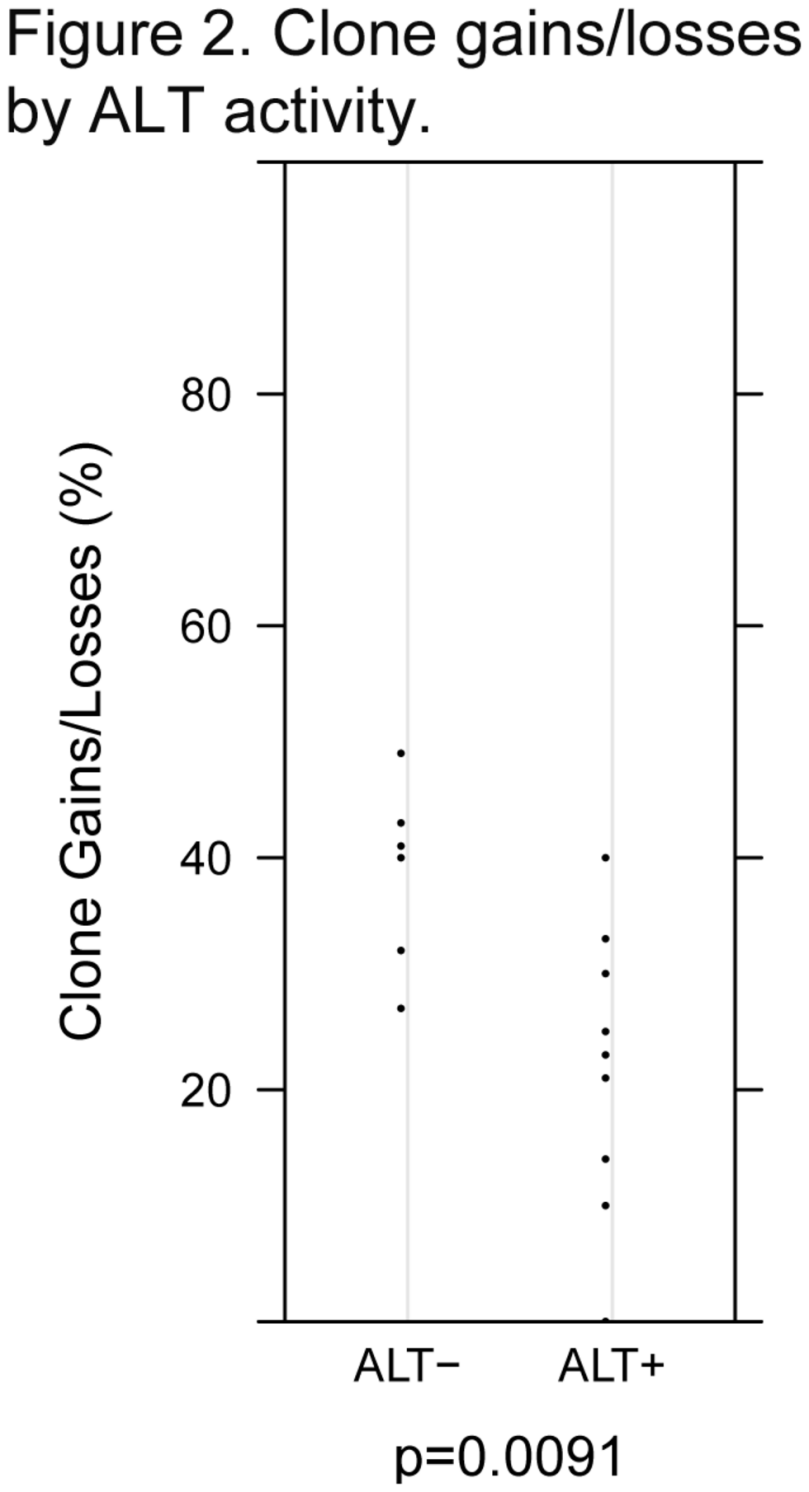
Clone gains/losses by ALT activity. Tumors that were ALT- exhibited significantly more chromosomal gains/losses than ALT+ rectal cancer (p=0.0091).

**Figure 3:**
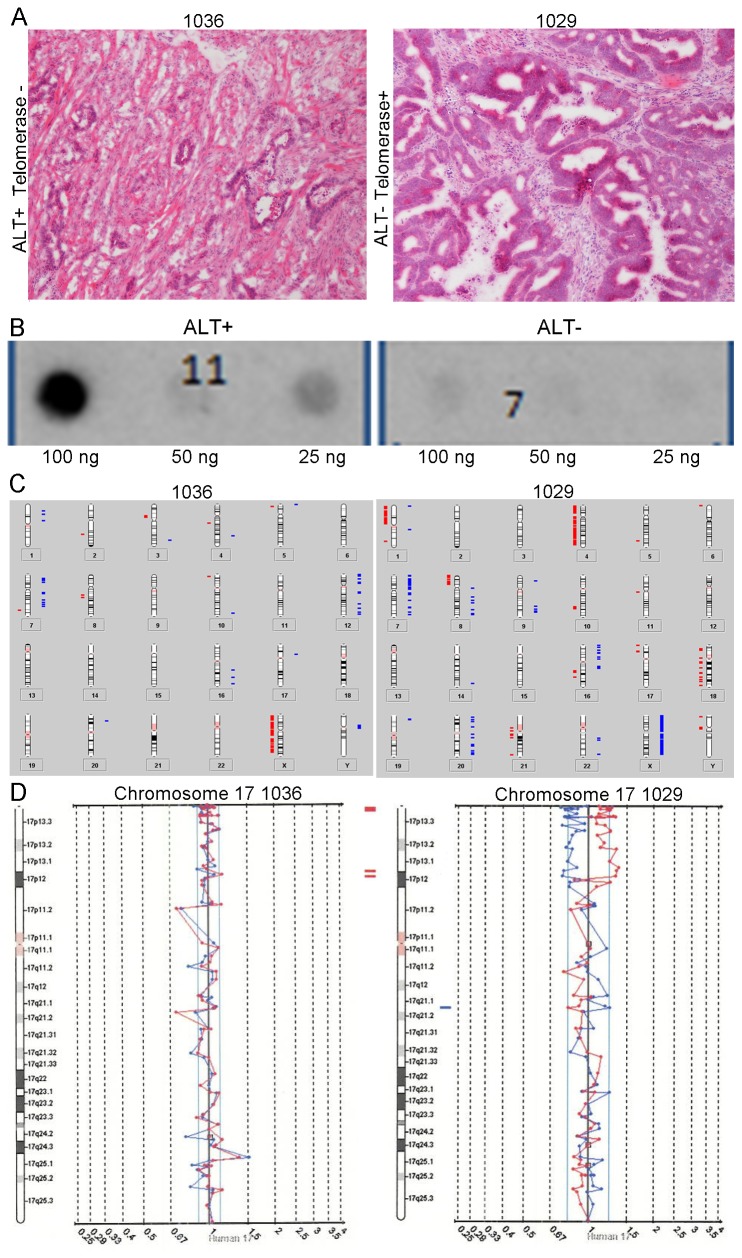
Histology, C-circle dot blot and aCGH summary for a MSS CIN- ALT + rectal cancer without activation of telomerase and MSS CIN+ ALT - rectal cancer with activation of telomerase. Panel A. Hematoxylin and Eosin tissue sections from an MSS CIN- , ALT+,Telomerase- rectal cancer (left) and from MSS CIN+, ALT-, Telomerase + rectal cancer. Both are moderately differentiated adenocarcinomas. The gland-to-stroma ratio is higher in the ALT+/tel- case, and it has less desmoplastic stroma. Panel B. Dot/blot showing presence of C-circles. C circles, extrachromosomal telomeric DNA, are strongly associated with ALT. Assessed in tumor DNA with isothermic amplification of C-circle complementary strand and hybridization with ^32^P-(CCCTAA)_3_ probe by Capital Biosciences (Capital Biosciences, Maryland, U. S. A. ), a sample was called ALT+ if C-circles were detected. The presence of C-circles are illustrated by the presence of radioactive tracer in the image on the left, and the absence of radioactivity in the blot on the right indicates absence of C-circles in the ALT- tumor. Panel C. Ideograms summarizing chromosomal gains and losses across all chromosomes evaluated by aCGH. The ALT+, telomerase negative tumor on the left had <10% of BAC clones showing aberrant hybridization and is classified as a CIN- tumor. The ALT-,,telomerase positive tumor on the right had 40% of clones with aberrant hybridization and is classified as a CIN+ tumor. Panel D. aCGH results of raw data for chromosome 17 for each tumor corresponding to the ideograms in Panel C.

We further evaluated whether differences in telomere length or CIN status may be related to activation of telomerase or ALT. We observed that tumors with shorter telomeres were more likely to have activation of telomerase (p=0.0040; Figure 1, Panel B) but tumor telomere length did not correlate with the presence of ALT (p=0.4923; Table 2). CIN+ tumors were more likely to show activation of telomerase than were CIN- tumors (p=0.0949; Table 1] but there was not a difference in gains/losses between CRC with telomerase activation and CRC without telomerase activation (p=0.3168; Table 1). The converse was true with regard to ALT. CIN- tumors were as likely as CIN+ tumors to show evidence of ALT (p=0.2335; Table 1) though those tumors that were ALT- had more gains/losses than ALT+ CRC (mean: 39.3 vs 22.6, p=0.0091; Figure 2, Table 2). Tumors that lengthened telomeres via exhibited C-Circle formation not present in ALT- tumors (Figure 3). 

**Table 2:** Telomere length and fraction of clone gains/losses by tumor characteristics.

	**Telomere Length**		**Fraction Gains/Losses**	
	**Mean (SD)**	**p-value**	**Mean (SD)**	**p-value**
CIN Status		0.0066		0.0007
CIN+	8211 (308)		33.1 (8.8)	
CIN-	9178 (1396)		9.7 (7.9)	
Telomerease		0.0040		0.3168
Yes	8193 (295)		28.8 (14.3)	
No	9307 (1512)		19.2 (16.4)	
ALT		0.4923		0.0091
Yes	8676 (1208)		19.6 (13.5)	
No	8226 (397)		38.7 (7.9)	
p53		0.5414		0.7726
Yes	8443 (370)		26.3 (17.0)	
No	8676 (1281)		22.9 (12.0)	

Rectal cancer tumors evaluated with array CGH were classified as 1) CIN- if <20% of clones showed DNA copy number changes or as 2) CIN+ if ≥ 20% of clones showed DNA copy number changes. Tumors with shorter telomeres were more likely to be CIN+ (p=0.0066) and to have activated telomerase (p=0.0040). Neither the presence of alternative lengthening of telomeres (ALT) (p=0.4923) nor the presence of p53 mutation status correlated with tumor telomere length (p=0.5414). CIN+ rectal cancer had a higher fraction of clones that showed gains and losses than CIN- tumors (p=0.0007). Tumors with or without activation of telomerase did not have a significantly different fraction of gains or losses of clones (p=0.3168). However, for tumors exhibiting ALT the fraction of gains and losses of clones was lower than that of tumors with no evidence ALT (p = 0.0091).No difference is seen in the fraction of clone gains or losses between tumors with our without p53 mutations (p=0.7726).

### p53 point mutations

Somatic p53 mutations were identified in 8 of 17 tumors evaluated by high phase liquid chromatography (dHPLC) with positive findings confirmed with direct sequencing. 

p53 mutations were not associated with CIN status (p=0.3348, Table 1), the clone % (p=0.7726, Table 2), the tumor telomere length (p=0.5414) or the telomerase or ALT activity of the tumor (p=1.00 and p=0.5804, respectively; data not shown). 

## Discussion

We previously reported that half of MSS CRC tumors from young onset (≤50 years of age) patients had diploid DNA chromosome content and may comprise a distinct subset of CRC that is both microsatellite and chromosomally stable [9]. Here we report that a subset of young onset MSS rectal cancers did indeed show low to no evidence of numerical chromosomal aberrations on aCGH, validating that a subset of MSS rectal cancer are CIN- on the basis of DNA copy number changes. We report that within CRC, subclasses of MSS rectal cancer tumors contain distinct molecular characteristics that include the presence of a subgroup of MSS rectal cancer that are CIN- and/or ALT+ and another subgroup that are CIN+ and have telomerase activation. Tumor telomere length and type of telomere maintenance distinguished MSS CIN- from CIN+ rectal cancer, indicating that these may be two divergent pathways to MSS rectal cancer. Though limited by the small sample size, this study reports on a molecular profile not previously recognized in MSS rectal cancer and indicates a need to examine this phenomenon more extensively.

Molecular subtyping of CRC has been proved important for individualized medical decision-making regarding best therapeutic options and may be used for prognostication. One published subtyping classification system utilized gene expression profiles [18] to determine response of CRC to cetuximab. De Sousa E Melo et al., combined genotyping and expression signatures to identify three molecular subtypes including 1) CIN+, 2) CpG island methylator phenotype (CIMP+), MSI(H) and 3) heterogeneous CIN and MSI and CIMP with no characterizable mutation pattern but a worse prognosis and expression patterns resembling those seen in sessile serrated adenoma and epithelial mesenchymal transition [19]. The molecular subtype we described in which a MSS tumor had little to no CIN but in which longer tumor telomeres and ALT were present may overlap with De Sousa E Melo’s subtype 3. More comprehensive evaluation of CRC characterized as CIN- and by its method of telomere mechanism may further our understanding of the relationship of MSS CIN- CRC and ALT with subtype 3. 

Tumor telomere attrition and its association with telomerase activation have been explained relative to the CIN+ status of the tumor DNA in the case that the checkpoints that normally initiate apoptosis and senescence are bypassed or inactivated, tolerance of severe telomere shortening can occur. Instead of triggering a response to terminate cells susceptible to malignant progression, these critically shortened telomeres persist, leading to chromosomal instability. Lacking proper length telomere caps, these cells are susceptible to developing end-to-end fusion, dicentric, multicentric and ring chromosomes as the extremely shortened telomeres stop functioning in their role to assist in the proper maintenance of a normal karyotype. Structural changes associated with CIN include unequal chromatin in segregated sister chromatids; anaphase bridging and multipolar mitoses. These structural changes can lead to changes in the number of chromosomes and loss of heterozygosity due to mitotically unstable chromosomes [20].

The sequence of events and implications of chromosomal stability relative to telomere elongation via ALT is not clearly defined in CRC since it has not been widely recognized to occur in this tumor type. ALT has been associated with breast cancer, osteosarcoma, and glioblastoma multiforme (GBM) [21]. ALT is distinct from telomerase associated telomere reconstitution; it is a recombination based mechanism that results in the creation of very long heterogeneous telomeres and ALT associated promyelocytic leukemia bodies (APBs) [22]. Polyploidization through whole genome duplication is a numerical increase in a whole set of chromosomes (i.e., triploidy or tetraploidy), and is newly recognized to be associated with ALT in cancers [23]. This is in contrast to telomerase associated numerical gains or losses in a particular chromosome(s) or parts of chromosomes called aneuploidy. 

ALT and telomerase may also occur simultaneously within a cancer and activation of telomerase has been noted in a small sampling of MSI(H) cancers [24]. In several small studies, the corresponding cancers were documented to have telomere lengths 3000 to 5000 bp shorter than the telomeres of the adjacent normal colon epithelium in up to 75% of CRC cases [25,26]. Among the CIN+ and telomerase activated tumors in our study, this same phenomenon was noted. In contrast, CIN- and ALT+ tumors, in our study featured telomere length longer than that of adjacent normal colon mucosa. Though not definitively proven, there is a large body of evidence suggesting that telomere shortening in the normal epithelium may be the precursor event that leads to the structural and numerical chromosome changes characteristic of many epithelial cancers and typical of CRC [27]. In our small sample size we were not able to generate a range of normal colon epithelium telomere length according to CIN phenotype to prove or disprove this, but for CIN+ tumors—which were more likely to activate telomerase—tumor DNA telomere length was up to 5 times shorter than that of the adjacent normal colon epithelium. For the CIN- tumors and those with ALT, the tumor DNA telomere length was significantly longer than that of the corresponding normal epithelium. 

 Though Chang et al. [28] found that MSS diploid tumors had a lower p53 mutation rate and MSS aneuploid tumors had a higher p53 mutation rate, an observation that has been independently confirmed [2], we did not detect an association of p53 mutations or LOH of p53 with CIN status, tumor telomere length or type of telomere maintenance repair utilized by the tumor. 

Controversy persists over the association of p53 mutations with tumor telomere length and cancer cell immortalization via telomere maintenance repair pathways. Both telomerase activation and ALT have been reported to function independently of p53 mutation [23], but some studies have determined that telomere dysfunction with associated telomerase activation may be more likely to intensify tumor promotion in the face of p53 mutation [29]. 

Whether the interconnection of a CRC’s CIN status with the tumor’s telomere length and the method of telomere maintenance repair of a MSS CRC impart a difference in the prognosis or responsiveness to treatment or long term disease outcome has not been addressed. Several studies that have included the category of MSS CIN- CRC determined that MSS CIN- CRC may be more aggressive [4,30]. The CIN status of CRC has been implicated as an independent factor for prognosis and multidrug resistance [31]. 

Telomerase activation has been associated with a worse prognosis for several cancer types, including rectal cancer [32]. Telomerase independent human rectal cancers not evaluated for ALT have been found to be more likely to have an earlier T stage (depth of invasion of the tumor through the rectal wall) but not N stage (locoregional lymph node involvement) or M stage (metastasis to distant organs). In SCID mice injected with ALT+, telomerase negative transformed mouse embryonic fibroblasts (MEF), the resultant cancerous subcutaneous tumors were unable to metastasize while ALT- telomerase activated MEFs were more aggressive, resulting in lung metastasis of telomerase positive cancers [33]. 

Prognostication in CRC classified by their CIN and telomere maintenance mechanisms may be developed as our understanding of the interactions of CIN and telomere dynamics grow. Telomere dynamics are complex, with evidence to suggest that though more than one type of telomere maintenance mechanism may be engaged either simultaneously or metachronously within the lifetime of a tumor [23], there may be an underlying predominant single mechanism of telomere maintenance driving an individual tumor’s course of progression. The development of telomerase inhibitors as experimental cancer treatment highlights the importance of characterizing a tumor based on activation of telomerase or ALT status [34,35]. 

To minimize heterogeneity related to tumor site or stage, we included only rectal cancers of stage B or C. Therefore, whether the association of MSS CIN- tumors with ALT and CIN+ tumors with telomerase activation will be similar in cancers arising in the colon or presenting at very early or later stage has yet to be evaluated. Though genetic profiling comparing colon to rectal cancer indicates that these tumors are genetically similar [36], assessment of CIN, ALT and telomerase activation in a larger sample size of both colon and rectal cancers is needed. MSI(H) colon or rectal cancers were not included in this study and the presence of ALT and/or telomerase activation—and less likely CIN—also require further study. 

Because CIN- and CIN+ CRC have distinctive clinical behaviors and molecular signatures, molecular classification of CRC may be enhanced if the telomere phenotype of a cancer is included in analyses of tumor molecular profiles, just as MSI phenotype of CRC is a useful molecular phenotypic classifier of CRC tumor behavior and the underlying pathway to colorectal carcinogenesis. Molecular classification of tumor maintenance mechanisms of MSS CRC on the basis of the CIN status, telomere length, and associated tumor telomere dynamics may impact prognostication and treatment options and provide a context for further study of telomere biology in CRC. Lastly, insight into the relationship between a tumor’s telomere length and regenerative dynamics coupled with chromosomal stability status may prove useful for the development of chemopreventive, early detection and treatment modeling to meet the needs of individual CRC patients. 

## Supporting Information

Figure S1 **Fractions of gains and losses of clones in CIN+ compared to CIN- colorectal cancers.** Colorectal cancer tumors evaluated with array CGH were classified as 1) CIN- if <1 to 17% of clones showed DNA copy number changes or as 2) CIN+ if ≥ 20% of clones showed DNA copy number changes. To establish CIN status based on chromosomal gains or losses, array comparative genomic hybridization (aCGH) data were analyzed in R. The fraction of participants with gains or losses of each clone was plotted by chromosome number and location for CIN- (panel A) and CIN+ (panel B). Differences in gains or losses occur throughout the genome, with the most pronounced differences between those classified as CIN- and CIN+ in chromosomes 13, 17 and 18.
(TIF)

Figure S2 **Percentage of either clones or tumors that show loss or gain for each chromosome arm.** Comparative genomic hybridization array (aCGH) results for MSS rectal tumors with corresponding peripheral blood leukocyte telomere length (PBL TL); normal colonic epithelium telomere length (nl TL); rectal cancer telomere length (ca TL); telomerase activation (telomerase) present (pos) or absent (neg); alternate lengthening of telomeres (ALT) determined by measurement of C-circles if C-circles present sample is ALT+ (pos) or absent ALT- (neg); p53 mutation (p53 mt), no mutation present (no) or mutation present (yes); tumor DNA ploidy by flow cytometry: aneuploidy (AN) or diploid (DNA); modified Astler-Coller tumor stage; B2 = tumor completely penetrates the smooth muscle layer into the serosa; C1 = tumor invades the muscularis propria with fewer than four positive nodes; C2 = tumor completely penetrates the smooth muscle layer into the serosa with four or more involved nodes; tumor recurrence (recur) yes or no. Six tumors (32%) had low levels of chromosomal disruption with < 20% of the.
(TIF)
