# Correction: Correlation of Chromosomal Instability, Telomere Length and Telomere Maintenance in Microsatellite Stable Rectal Cancer: A Molecular Subclass of Rectal Cancer

**DOI:** 10.1371/journal.pone.0102207

**Published:** 2014-07-01

**Authors:** The PLOS ONE Staff

In Figure 3A, the two images are reversed. Please see the correct Figure 3 here.

**Figure 3:**
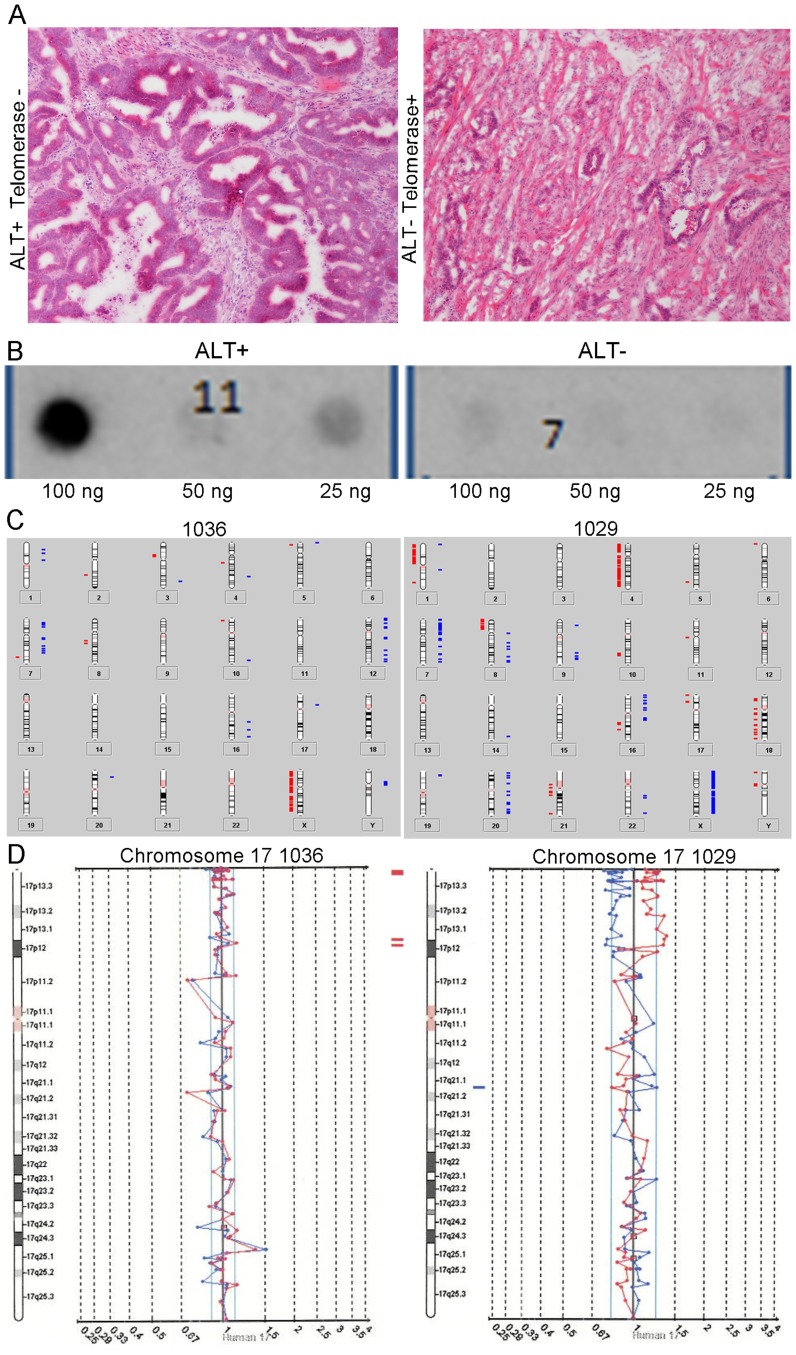
Histology, C-circle dot blot and aCGH summary for a MSS CIN- ALT + rectal cancer without activation of telomerase and MSS CIN+ ALT - rectal cancer with activation of telomerase. Panel A. Hematoxylin and Eosin tissue sections from an MSS CIN- , ALT+, Telomerase- rectal cancer (left) and from MSS CIN+, ALT-, Telomerase + rectal cancer. Both are moderately differentiated adenocarcinomas. The gland-to-stroma ratio is higher in the ALT+/tel- case, and it has less desmoplastic stroma. Panel B. Dot/blot showing presence of C-circles. C circles, extrachromosomal telomeric DNA, are strongly associated with ALT. Assessed in tumor DNA with isothermic amplification of C-circle complementary strand and hybridization with ^32^P-(CCCTAA)_3_ probe by Capital Biosciences (Capital Biosciences, Maryland, U. S. A. ), a sample was called ALT+ if C-circles were detected. The presence of C-circles are illustrated by the presence of radioactive tracer in the image on the left, and the absence of radioactivity in the blot on the right indicates absence of C-circles in the ALT- tumor. Panel C. Ideograms summarizing chromosomal gains and losses across all chromosomes evaluated by aCGH. The ALT+, telomerase negative tumor on the left had <10% of BAC clones showing aberrant hybridization and is classified as a CIN- tumor. The ALT-,,telomerase positive tumor on the right had 40% of clones with aberrant hybridization and is classified as a CIN+ tumor. Panel D. aCGH results of raw data for chromosome 17 for each tumor corresponding to the ideograms in Panel C.

## References

[1] Boardman LA, Johnson RA, Viker KB, Hafner KA, Jenkins RB, et al (2013) Correlation of Chromosomal Instability, Telomere Length and Telomere Maintenance in Microsatellite Stable Rectal Cancer: A Molecular Subclass of Rectal Cancer. PLoS ONE 8(11): e80015 doi:10.1371/journal.pone.0080015 24278232 10.1371/journal.pone.0080015 PMC3836975

