# Supplementary figures and images for: Correlation of Chromosomal Instability, Telomere Length and Telomere Maintenance in Microsatellite Stable Rectal Cancer: A Molecular Subclass of Rectal Cancer
Source: PLoS One. 2013 Nov 21;8(11):e80015. doi: 10.1371/journal.pone.0080015 (PMC3836975; doi:10.1371/journal.pone.0080015)

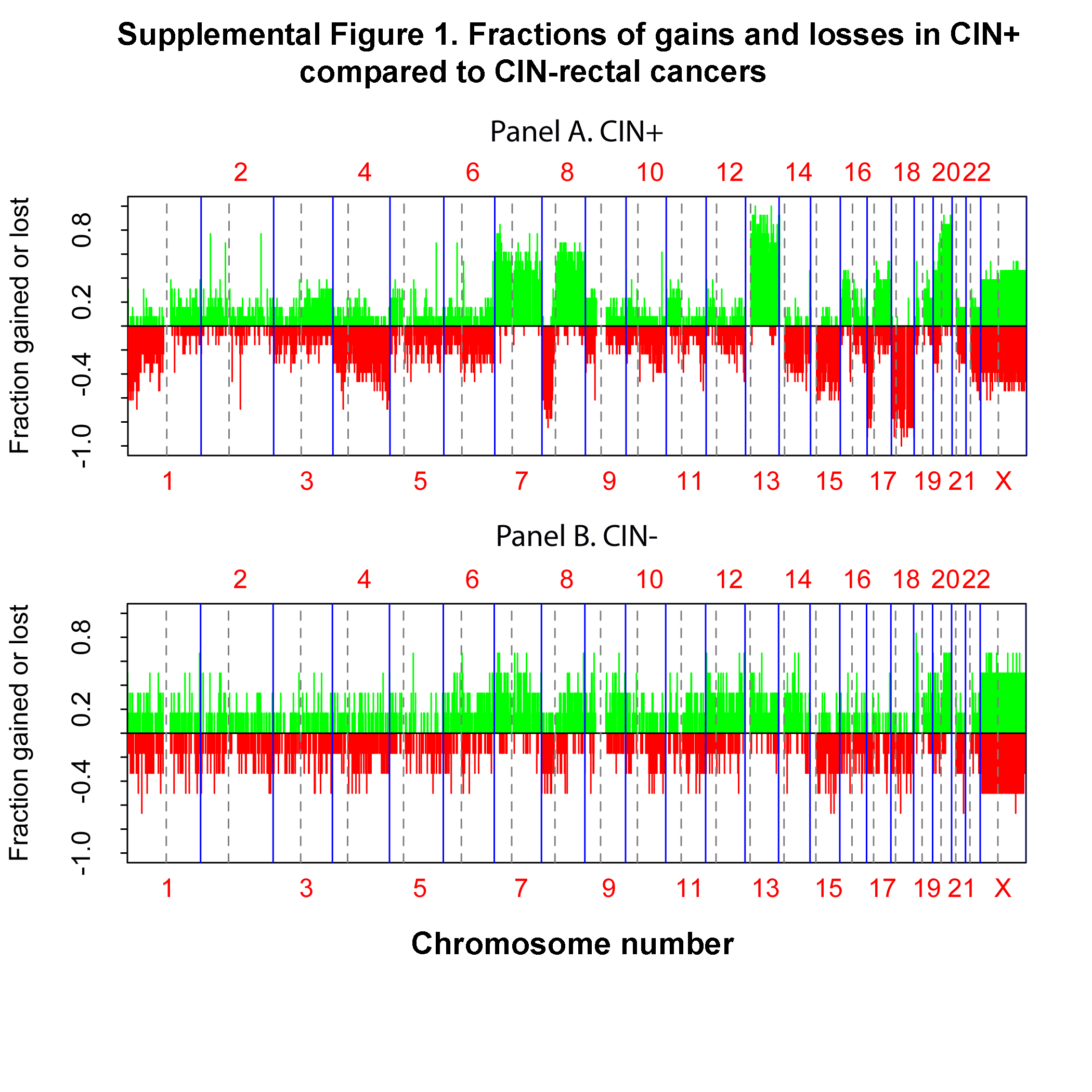

Supplement: Figure S1 — Fractions of gains and losses of clones in CIN+ compared to CIN- colorectal cancers. Colorectal cancer tumors evaluated with array CGH were classified as 1) CIN- if <1 to 17% of clones showed DNA copy number changes or as 2) CIN+ if ≥ 20% of clones showed DNA copy number changes. To establish CIN status based on chromosomal gains or losses, array comparative genomic hybridization (aCGH) data were analyzed in R. The fraction of participants with gains or losses of each clone was plotted by chromosome number and location for CIN- (panel A) and CIN+ (panel B). Differences in gains or losses occur throughout the genome, with the most pronounced differences between those classified as CIN- and CIN+ in chromosomes 13, 17 and 18. (TIF) [file pone.0080015.s001.tif]

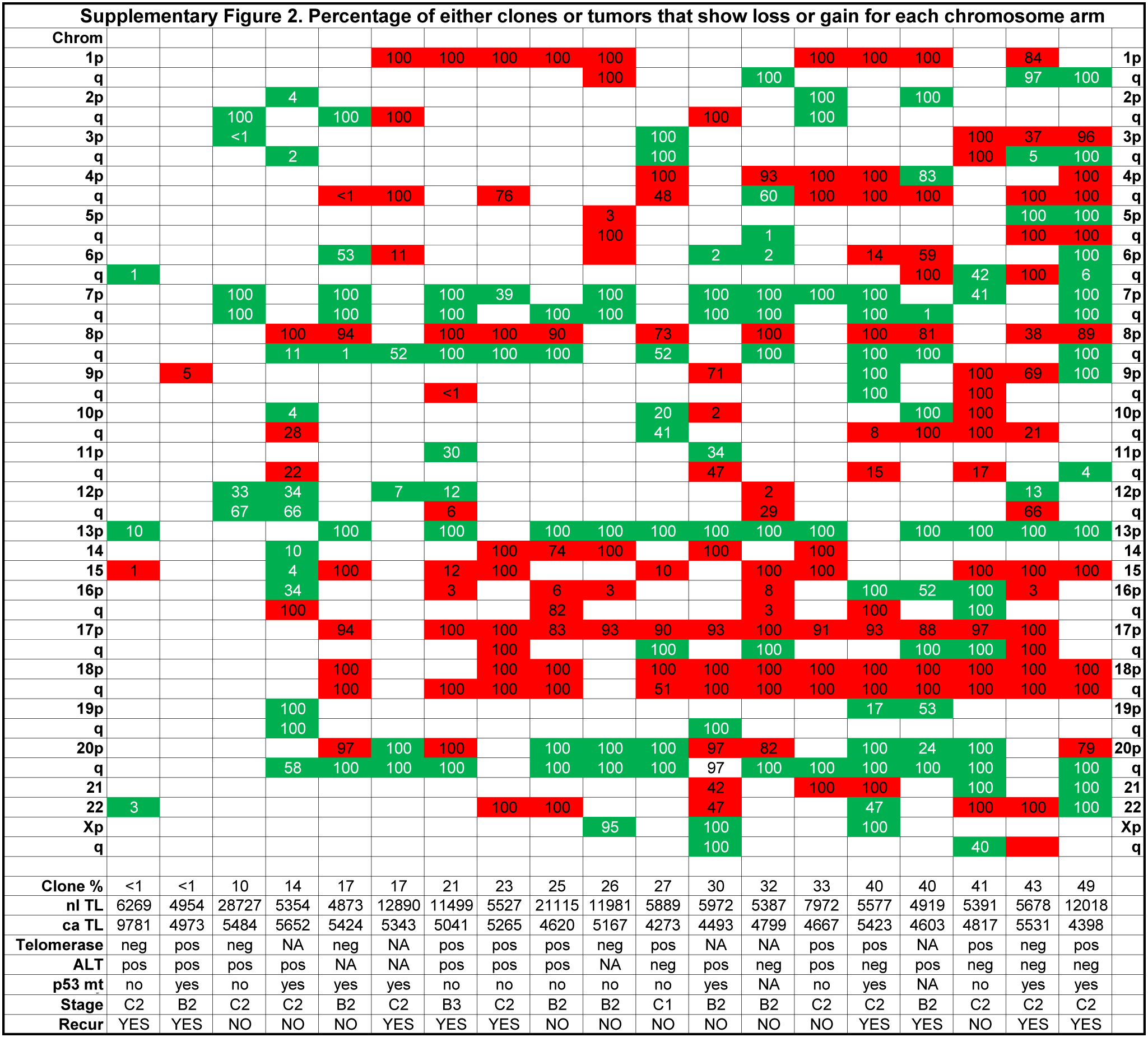

Supplement: Figure S2 — Percentage of either clones or tumors that show loss or gain for each chromosome arm. Comparative genomic hybridization array (aCGH) results for MSS rectal tumors with corresponding peripheral blood leukocyte telomere length (PBL TL); normal colonic epithelium telomere length (nl TL); rectal cancer telomere length (ca TL); telomerase activation (telomerase) present (pos) or absent (neg); alternate lengthening of telomeres (ALT) determined by measurement of C-circles if C-circles present sample is ALT+ (pos) or absent ALT- (neg); p53 mutation (p53 mt), no mutation present (no) or mutation present (yes); tumor DNA ploidy by flow cytometry: aneuploidy (AN) or diploid (DNA); modified Astler-Coller tumor stage; B2 = tumor completely penetrates the smooth muscle layer into the serosa; C1 = tumor invades the muscularis propria with fewer than four positive nodes; C2 = tumor completely penetrates the smooth muscle layer into the serosa with four or more involved nodes; tumor recurrence (recur) yes or no. Six tumors (32%) had low levels of chromosomal disruption with < 20% of the. (TIF) [file pone.0080015.s002.tif]
